# How Cognitive Reserve should Influence Rehabilitation Choices using Virtual Reality in Parkinson's Disease

**DOI:** 10.1155/2022/7389658

**Published:** 2022-09-16

**Authors:** Letizia Pezzi, Andrea Di Matteo, Roberta Insabella, Sara Mastrogiacomo, Carlo Baldari, Victor Machado Reiss, Teresa Paolucci

**Affiliations:** ^1^Rehabilitation Unit, ASST Cremona–Ospedale di Cremona, Cremona, Italy; ^2^Department of Oral and Biotechnological Biomedical Sciences, Physical Medicine and Rehabilitation, University of G. D'Annunzio of Chieti-Pescara, Chieti, Italy; ^3^Department of Anatomical and Histological Sciences, Legal Medicine and Orthopedics, Sapienza University of Rome, Rome, Italy; ^4^Fondazione Santa Lucia IRCCS, Rome, Italy; ^5^Department of Theoretical and Applied Sciences, University eCampus, Novedrate, Italy; ^6^Research Center for Sport, Health, and Human Development, University of Tras-Os-Monte and Alto Douro, Vila Real, Portugal

## Abstract

Virtual reality (VR) is used in the rehabilitation of patients with Parkinson's disease (PD) in several studies. In VR trials, the motor, physical characteristics, and the degree of the disease are often well defined, while PD cognitive reserve is not. This systematic review was performed to define a cognitive profile for patients with PD who could best benefit from using VR to enhance functional motor aspects during rehabilitation. PubMed, Cochrane Library, Scopus, and Web of Sciences databases were analysed to identify randomized clinical trials (RCT) and randomized pilot trials that addressed the rehabilitation of motor symptoms in subjects with PD using VR. The included studies used Mini-Mental State Examination (MMSE) or Montreal Cognitive Assessment (MoCA) to evaluate the cognitive aspect. Only articles written in English and with full texts were considered. The risk of bias from all included studies was assessed based on the Cochrane risk-of-bias tool and the PRISMA guideline was considered. Eighteen articles were eligible for review, including three randomized pilot trials. All studies aimed to evaluate the effect of VR on the motor aspects typically affected by PD (balance, postural control, risk of falls, walking, and reaching). The most widely adopted approach has been nonimmersive VR, except for one study that used immersive VR. Both the benefits of physical activity on the motor symptoms of patients with PD and the impact of cognitive reserve during the rehabilitation of these patients were highlighted. The analysis of the results allowed us to outline the ideal cognitive profile of patients with PD who can benefit from the effects of rehabilitation using VR.

## 1. Introduction

Parkinson's disease (PD) is a chronic progressive neurodegenerative disease, characterized by the loss of dopaminergic neurons in the pars compacta of the substantia nigra and the accumulation of alpha-synuclein aggregates in specific regions of the brain stem, spinal cord, and cerebral cortex [1]. The estimated prevalence of PD in industrialized countries is 0.3% in the general population (1% in people over the age of 65 and 3% over 80 years), with incidence rates from 11 to 19 per 100,000 people each year [2, 3].

Patients with PD may also have affected cognitive functions, particularly global cognitive performance (as measured by the MMSE screening test) and behavioral deficits that affect aspects of social and community life [4]. The most common cognitive symptoms are deficit of attention and executive functions (working memory, planning, and inhibition), difficulties in episodic memory, verbal fluency, and visuospatial and visuoperceptual abilities [5].

Cognitive reserve (CR) is a theoretical construct that describes differences in individuals' susceptibility to cognitive, functional, or clinical decline due to ageing or neurological disease [6]. This concept is fundamental in neurodegenerative disorders such as PD, considering the severity of motor and cognitive disability and the functional impact on daily life [6]. Higher levels of CR are thought to be related to delayed disease onset and higher cognitive performance [7], and higher CR was associated with a better performance on the MMSE, thus confirming the protective role of CR on global cognitive functioning.

CR cannot be measured directly; it encompasses several different factors, including genetics, environment, education, occupational demands, lifetime experiences, and mental stimulation [8, 9].

Studies in the literature show that the level of education and physical activity, especially aerobic, and cognitive activities, reducing the loss of brain mass and strengthening compensatory circuits, have protective effects on the brain [10].

The study by Koerts et al. highlighted the relationship between CR and impairment of executive functions, that is, cognition skills; they pointed out that patients with PD who have high premorbid intellectual capacity show fewer cognitive deficits than patients with low premorbid capacity [11].

The complex management of PD can be achieved through a calibrated combination of drug therapy and rehabilitation. Physiotherapy aims to maximize the quality of movement and promote functional independence and general fitness in patients with PD, minimizing secondary complications of the disease [12].

From a rehabilitation perspective, virtual reality (VR) represents an alternative, noninvasive therapeutic modality, often used in association with conventional rehabilitation, to cope with the degenerative characteristics of PD. Furthermore, VR is more captivating for patients with high CR, as Pazzaglia et al. pointed, and the therapeutic exercise is perceived as more exciting and fun by having visual and auditory feedback contextual to the movement [13].

The following are the two main categories of VR: immersive, which allows a more direct experience of virtually generated environments, and nonimmersive, which allows a subject to observe, through a standard high-resolution monitor, a virtual environment with which he/she can interact through interfaces, such as keyboards and controllers [14].

Various studies, considering the premises to integrate cognitive and motor aspects, propose multimodal rehabilitation approaches that combine motor training with cognitive stimuli through technologies and virtual reality for patients with PD. The common goal is to create an enriched environment capable of stimulating different cognitive aspects, involving the subjects with a more playful approach [15–17].

Often, the physical characteristics and disease stage of PD patients, included in VR trials, are well defined; the same does not happen regarding their cognitive profiles. This could erroneously suggest that VR-associated motor rehabilitation may be useful to all patients with PD regardless of the degree of cognitive reserve. About 25% of patients with PD, especially after the age of 70, may experience mild cognitive impairment or dementia. In addition, in patients with PD, dual-task rehabilitation exercises or multimodal activities are not always recommended, especially in the presence of cognitive or complex tasks, which can lead to freezing of gait, loss of balance, and increased falls, all due to attention deficits and the reduction of automatisms and psychomotor speed in patients with PD [18].

From this premise, our hypothesis was that VR may be effective in patients with PD who respond to a specific neuro-cognitive profile. On the other hand, in PD patients with inadequate cognitive reserve, VR may not have the same therapeutic efficacy as suggested by Imbimbo et al., where patients with a higher cognitive reserve benefited more from the VR treatment. In contrast, patients with low cognitive reserve could achieve better results by following a traditional rehabilitation program [19].

Therefore, the cognitive reserve of PD patients could indicate the disease's evolution and help clinicians choose the most suitable rehabilitation strategy [14].

As a result, the considerations addressed so far lead to the goal of this systematic review, which is investigating to what extent neuro-motor rehabilitation with VR is useful for improving the motor aspects in patients with PD in relation to the cognitive reserve.

## 2. Materials and Methods

### 2.1. Search Strategy

This systematic review included articles published in the last 10 years (from 2011 to July 2021), according to the Preferred Reporting Items for Systematic Reviews and Meta-Analysis (PRISMA) guidelines [20], and evaluated studies related to the rehabilitation of patients with PD using VR in the following databases: PubMed, Cochrane Library, Scopus, and Web of Science.

Different combinations of the following MeSH terms were used to select the articles: (Parkinson OR Parkinson's Disease) AND virtual reality AND (rehabilitation OR training OR exercise).

The reference lists for most of the relevant studies were scanned for additional citations. Country, author, affiliated institution, and enrolment period data were extracted and reviewed to identify and exclude duplicate publications using the same cohort. Any disagreement regarding accepting full-text articles was resolved by discussion until a consensus was reached.

### 2.2. Study Eligibility Criteria

Our target was randomized clinical trials (RCTs) and randomized pilot studies (full text in English) and studies evaluating cognitive aspects using the Mini Mental State Examination (MMSE) [21] or Montreal Cognitive Assessment (MoCA) [22] and that deal with the motor aspects of rehabilitation through virtual reality; the motor aspects were “balance,” “falls,” “ambulation,” “postural control,” and “reaching.” MMSE is a short exam, used to evaluate the patient's neuro-cognitive performance by administering a few questions to test orientation, memory, attention, calculation, and language. The total score is between 0 and 30; a score ≥ 24 indicates normal values. The MoCA acts as a quick screening for mild cognitive impairment. It evaluates different cognitive domains: attention and concentration, executive functions, memory, language, visuo-constructive skills, abstraction, calculation, and orientation. The maximum possible score is 30; a score ≥26 is considered normal.

Studies that adopted one or both of the previously mentioned scales were preferred to facilitate the analysis of the results. In this way, based on similar and comparable data, it was possible to consider the cognitive characteristics of the patients.

Studies other than RCTs and those that administered VR treatment to patients with neurological conditions other than PD (stroke and multiple sclerosis) were excluded.

### 2.3. Studies Quality Evaluation

The methodological quality of the studies was assessed using the PEDro scale [23]. Studies with scores ≥ 9 were of “excellent” quality. Studies with scores from 6 to 8 were considered “good,” studies with scores from 4 to 5, “fair,” and those with scores ≤4, “poor.” The risk of bias was also assessed for each RCT using the Cochrane risk-of-bias tool [24]. The main domains were evaluated in the following sequence: (1) selection bias (generation of randomized sequences and allocation concealment); (2) reporting bias; (3) performance bias (blindness of participants and staff); (4) detection bias (blinding of the evaluation of results); (5) attrition bias (incomplete outcome data, such as those due to dropouts); (6) other sources of bias. The scores for each domain of bias and the final score for the risk of systematic bias were classified as low, high, or unclear risk.

## 3. Results

**Figure 1:**
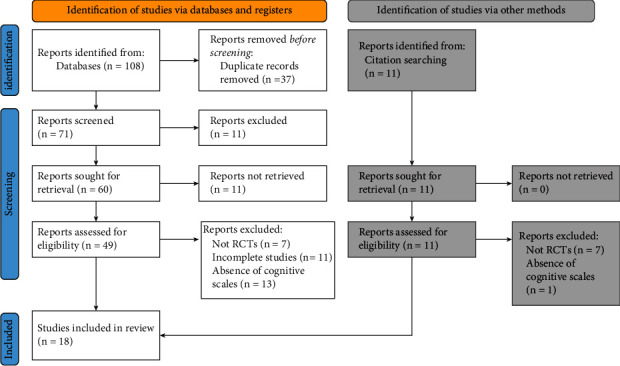
PRISMA flow-diagram.

The initial search, carried out through electronic databases, produced 108 results. This search was complemented by a manual search of individual citations of systematic reviews and articles included in the review, identifying 11 additional studies. After duplicates were removed, the remaining 71 publications were reviewed according to their titles and abstracts. This led to the exclusion of 11 publications. Of the remaining 60, the full text of 11 studies could not be found (as they were posters and abstracts presented at conferences). The subsequent screening of the remaining complete texts allowed the identification of 15 publications relevant for the revision, and another three studies resulting from the selection of citations were added. Overall, 18 RCTs published in English were screened for inclusion (Figure 1).

The selected studies are shown in Table 1, which describes the type of VR and the protocols used, the results measured, the evaluation times, and the presence of adverse events during treatment.

According to the PEDro scale (Table 2), the mean methodological quality of the included RCTs was 6.1, indicating the overall good quality of the included studies. The risk of bias was considered low for 11 articles, while for the remaining seven articles, it was considered high (Table 3). The most frequent sources of potential bias were performance bias (related to participant and staff blinding), concealment of distribution in groups, the presence of uncompensated dropouts from analysis by intention to treat, and incomplete result data.

It should be noted that, among the studies included in the review, Del Din et al. [26], Mirelman et al. [35], and Pelosin et al. [36] included elderly patients with mild cognitive impairment; therefore, of the 1393 patients analysed in the studies, only 1052 were PD patients, with a mean age of 68.8 years and a mean disease duration of 8.47 years.

**Figure 2:**
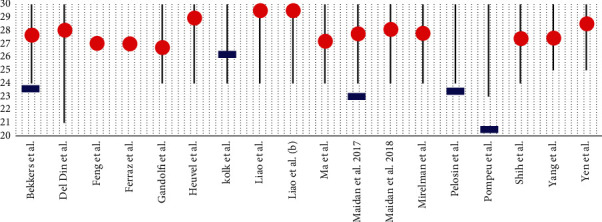
The mean of cognitive reserve score for each study included in the review. The grey line indicates the distribution of MMSE scores utilized as inclusion criteria for enrolled patients. The red spot indicates the mean MMSE score, and the blue tag, the mean MoCa score.

**Figure 3:**
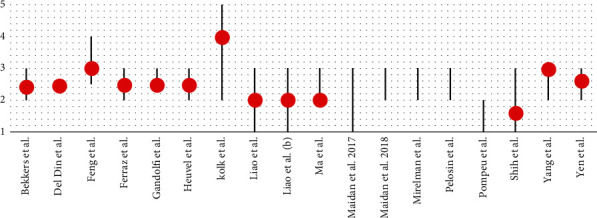
The grey line indicates the distribution of H&R scores of patients enrolled in the trials, and the red spot indicates the mean score of the H&R scale of patients who participated in the VR group.

In all studies, the cognitive level of the patients was assessed using the MMSE and/or the MoCA. The inclusion of patients in the trials, except in Del Din et al.'s study [26], was defined using the Hoehn and Yahr scale (H&Y) [41] relating to the progression of the disease (Figures 2 and 3).

A synthesis study was conducted on the patients included in the various protocols to observe under which conditions VR can be used effectively. None of the studies examined showed the influence of cognitive reserve (or the analysis of the patient's cognitive profile) on the results. However, no study planned the treatment protocol with VR by comparing subjects with high cognitive reserve and groups with poor cognitive reserve.

All the studies included in the review aimed to evaluate the effects of VR-associated rehabilitation on the motor characteristics typically affected by PD. More specifically, seven trials [25, 27–29, 31, 37, 39] focused on improving balance; five studies [15, 25, 26, 35, 36] addressed the risk of falls reduction and their incidence.; seven articles [17, 26–28, 32, 34, 39] aimed to evaluate the effects of rehabilitation treatment on walking; three articles [28, 38, 40] dealt with the problem of postural control in patients with PD; only one article [30] focused on motor symptoms (assessed through the third section of the UMPRS scale) and one article [33] dealt with motor performance during reaching exercises. Unfortunately, it is impossible to compare the individual studies' results as different outcome measures were used.

Among the studies listed, two [32, 40] evaluated the effects of VR on sensory integration, and three [16, 34, 36] examined the effect of VR on brain activation and cholinergic activity.

All studies adopted nonimmersive virtual reality systems [16, 25–32, 34–37, 39, 40] or exergaming [17, 38], except for one study [33], which used immersive VR.

## 4. Discussion

This review aims to investigate to what extent neuromotor rehabilitation with VR is useful for improving the motor aspects in PD patients in relation to the cognitive reserve.

Most of the studies analysed in this review stated that VR associated with conventional rehabilitation produced better results, compared with rehabilitation alone, in terms of increasing motor characteristics, such as walking, balance. and postural stability, typically affected by PD. VR is a good rehabilitation option, especially when combined with conventional therapy, and seems more suitable in patients with a good cognitive reserve, measured indirectly with mean MMSE and MoCA scores of 27.94 ± 0.86 and 23.43 ± 2.04, respectively. Working with VR can be stimulating in patients with a high cognitive reserve as it is challenging, as Pazzaglia et al. pointed, where the exercises are perceived as interesting, motivating, and funny, providing immediate visual and auditory feedback [13].

In the literature, several studies consider VR an efficient tool for the rehabilitation of patients with PD. Endurance training, especially exercises performed on the treadmill, can improve balance, reduce gait disturbances, improve speed, stride length, and walking [42–44]. VR offers the opportunity to simulate immersive and controllable environments, with the possibility of customizing the rehabilitation treatment.

However, within the review, there are several articles, eight specifically [17, 25, 29, 32, 33, 37, 39, 40], which considered the effects of rehabilitation associated with VR on a par with those of conventional rehabilitation, suggesting that the use of VR could complement rehabilitation to increase motivation during treatment [17]. The home-based administration of VR could represent a valid alternative for subjects with PD with limited access to rehabilitation services [39].

Examples of exercises with VR carried out at home can be found in the studies of Gandolfi et al. [28] and van der Kolk et al. [30], which have associated VR with balance training and aerobic exercise, respectively.

It should be emphasized that the activity performed through VR guarantees good adherence to the treatment because, during the exercise, the integration of motor and cognitive skills is favored and reward circuits of the brain are stimulated [28, 45], which increases the possibility for patients to choose to train at any time of the day.

However, it is necessary to consider the cognitive aspects of patients with PD and their complex motor picture. Gandolfi et al. [28] admit that their results study should not be generalized and applied in patients with significant cognitive decline, as VR could be risky. Indeed, the European Physiotherapy Guideline for Parkinson's disease [18] says that dual-task or multimodal therapeutic exercises are not always indicated for parkinsonian patients because they can lead to freezing, loss of balance, and increased falls, especially during complex cognitive tasks. For this reason, VR could only be used with patients that respond to a specific cognitive profile since it may not have the same therapeutic efficacy in others with reduced cognitive reserve.

This is confirmed by the study of van der Kolk et al. [30], in which the participants' cognitive level, considered normal (MoCA = 26, 3), did not prevent the occurrence of adverse events, such as arthralgia, back pain, and palpitations, related to VR treatment, as well as falls, heart problems, and musculoskeletal damage, even if not related to exercise. In addition, the same authors state that several patients, from the VR treatment group, have left the study due to the onset of technical problems; this suggests that the presence of supervision or assistance can help in some circumstances to continue a training session with VR.

Although VR shows numerous advantages (related to learning motor skills through repetitive practice, performance feedback, and motivation) [46], it also presents some critical issues for patients: insufficient perception of depth and lack of tactile feedback (which, the latter, can cause difficulties when performing virtual tasks that simulate reality) [47, 48]. In addition, a recent systematic review results state that patients with advanced age may find VR games complicated or boring and may need supervision to complete the task undertaken [49].

Some of the studies described using VR rehabilitation programs have shown how resistance exercises, stretching, and cognitive rehabilitation can improve the patient's quality of life. This is because patient perceives themselves as an active part of treatment. However, the cognitive reserve was not considered, and its impact on rehabilitation was therefore not evaluated. However, we can infer from the review studies and the literature that the cognitive reserve should be considered in the evaluation phase of patients with PD to plan the optimal, tailored therapeutic approach.

Piccinini et al. [50] examined the influence of cognitive reserve on balance rehabilitation, using conventional therapy, in patients with PD. The results showed an improvement in balance, and regarding the relationship between the cognitive reserve and balance, the condition of patients with a lower cognitive reserve index (those with a lower level of education) improved more than that of patients with a high cognitive reserve index. It has been hypothesized that patients with better cognitive reserve should work on more stimulating mental tasks through approaches such as VR, dance, and technological tools. They found an inverse correlation between the level of cognitive reserve and the improvement of balance in patients with PD undergoing traditional rehabilitation, which highlights the important role that life experience, education, and recreational activities play on the individual's ability to cope with a brain pathology.

Imbimbo et al. [19] examined the relationship between VR and the cognitive reserve in patients with PD. The exercises proposed were intended to improve coordination and balance. At the end of the study, it was observed that, in relation to the cognitive reserve, some patients, unlike others, showed no improvement. VR showed better result in patients with a medium/high cognitive reserve index; these PD patients are accustomed to the use of technology, unlike subjects with a lower level of cognitive reserve who were uncomfortable with this tool and may felt less stimulated to learn. The results of our review may confirm Piccinini et al.'s results [50], which had suggested using a more complex rehabilitation approach for patients with a higher cognitive reserve.

This study had a few limitations: only four databases were searched, and we acknowledge the possibility that we did not identify all relevant studies.

## 5. Conclusion

Most of the studies analysed in this review included subjects with an MMSE score ≥ 24 and a H&Y stage between 2 and 3. Rehabilitation associated with VR was proposed for patients with PD with a mean score (mean of averages) of 27.94 (SD = 0.86) and 23.43 (SD = 2.04) for MMSE and MoCA, respectively, which shows a normal or slightly reduced cognitive level (if we consider the cut-off of 26 for MoCA).

According to the disease progression state, patients with PD who underwent treatment with VR had, on average (average of averages), an H&Y stage of 2.5 (SD = 0.60), indicating a slight bilateral involvement of the disease with recovery of balance on the pull test.

In conclusion, the results of these studies show that VR is a useful strategy that improves motor aspects mainly affected by PD and is feasible for patients with a normal cognitive level and an H&Y's stage less than three. This innovative approach, excluding excessively strenuous activities, is feasible at home and should preferably be performed in the presence of a caregiver or supervision.

## Figures and Tables

**Table 1:** Characteristics of the included studies.

Author, year, Title	Study type, PEDro score	Participants	VR type	Device/software/tools/protocol	Inclusion and exclusion criteria	Adverse events	H&Y score	Cognitive scales	Other outcomes	Evaluation times	Objective of the study	Conclusions
Bekkers et al., 2020 [25] Do patients with Parkinson's disease with freezing of gait respond differently than those without to treadmill training augmented by virtual reality?	RCT, 6	No: 121 (FOG+ = 77, FOG− = 44) VRG: 62 TG: 59 Age VRG: 71.06 TG: 70.86 Disease duration, years VRG: 9.05 TG: 9.55	Nonimmersive VR	VRG: treadmill with simulated obstacles in a virtual environment TG: treadmill Both treatments, lasting 45 minutes, were carried out 3 times a week for 6 weeks	I.C: age 60–90; H&Y II-III; anti-Parkinson therapy; walking for 5 minutes without assistance; adequate vision and hearing; 2 or more falls in the previous 6 months E.C: psychiatric comorbidities; MMSE <24; history of stroke, head injury, or other neurological disease other than PD; orthopaedic or rheumatic diseases; acute lower back pain or pain in limbs, peripheral neuropathy, inability to participate in training.	—	VRG: 2.42 TG: 2.49	MMSE: VRG = 27.76 (1.7) TG = 28.34 (1.5) MoCA: VRG = 23.85 (4.5) TG = 24.27 (3.5)	Primary: balance and falls: mini-BEST Secondary: number of falls NFOG-Q TMT-B SPPB FSST FES-I PASE MMSE MoCA UPDRS-III	T0 (baseline); T1 (after 1 week); T2 (after 6 weeks) T3 (6 months follow up)	To show a reduction in falls and improvement in balance following training on the treadmill with virtual reality compared to the treadmill alone, highlighting any differences between patients with and without FOG.	FOG patients improved balance and risk of falls in treadmill training both with and without VR compared to non-FOG patients
Del Din et al., 2020 [26] Falls risk in relation to activity exposure in high-risk older adults	RCT, 5	No tot = 282, PD = 128 Age 71.68	Nonimmersive VR	VRG: treadmill associated with VR in elderly patients/with MCI/PD TG: treadmill Both treatments, lasting 40 minutes, were carried out 3 times a week for 6 weeks.	I.C: age 60–90; able to walk for 5 minutes without assistance; stable therapy the month before; 2 or more falls in the previous six months E.C: psychiatric comorbidities; history of stroke, brain damage and other neurological disorders; acute lower back pain; rheumatic or orthopaedic diseases; MMSE <21	—	48%: 2 10%: 2.5 42%: 3 Mean MP = 2.47	MMSE (MP) 28.07 (1.68)	FRA index (Measure of the incidence of falls corrected for exposure)	T0 (pre-treatment); T1 (after 1 week) T2 (after 1 month) T3 (after 6 months)	To study the relationship between gait (exposure to falling risk) and fall rates before and after a treadmill exercise program with and without VR (V-TIME)	V-TIME intervention successfully reduced the risk of falling by maintaining walking activity levels in different groups of elderly people at risk of falling
Feng et al., 2019 [27] Virtual reality rehabilitation versus conventional physical therapy for improving balance and gait in Parkinson's disease patients: a randomized controlled trial	RCT, 7	No: 28tot VRG: 14 TG: 14 Age VRG: 67.47 TG: 66.93	Nonimmersive VR	VRG: perform game-based actions with a standard VR device TG: conventional treatment 45 treatment, once a day, 5 times a week for 12 weeks	I.C: H&Y 2.5/4; age 50–70; signed informed consent E.C: other causes of tremor; severe bone or joint disease; visual or hearing disturbances; inability to cooperate in the study	—	VRG: 3.03 TG: 2.97	MMSE: VRG = 27.07 ± 2.09; TG = 26.29 ± 2.49 Education received (years) VRG: 10.47 TG: 9.93	BBS TUG UPDRS III FGA	T0 (baseline) T1 (after 12 weeks)	Studying the effects of virtual reality on balance and walking in patients with PD	Rehabilitation with VR improved outcomes compared to traditional treatment (except for UPDRS where no significant differences were noted)
Ferraz et al., 2018 [17] The effects of functional training, bicycle exercise, and exergaming on walking capacity of elderly patients with Parkinson disease: a pilot randomized controlled single-blinded trial	Pilot RCT, 7	No: 62 TG1: 22 TG2: 20 VRG: 20 Age: TG1: 71 TG2: 67 VRG: 67 Disease duration, years: G1: 4 G2: 6 G3: 4	Exergame (nonimmersive VR)	VRG: training with “Kinect Adventures” video game (Xbox 360) TG1: functional training TG2: cycling exercise. 50′ treatment, 3 times a week, for 8 weeks	I.C: age ≥ 60; regular use of therapy; H&Y II-III walking without aids E.C: visual and hearing impairment; parkinsonian syndromes other than PD; orthopaedic diseases limiting physical activity; chronic uncontrolled diseases; cardiovascular disease; use of alcohol or toxic substances; contraindications to physical activity; practiced physical activity programs in the past 6 months; or participate in endurance training in the previous 12 months	—	TG1: 2.50 (2.5–3) TG2: 2.50 (2-3) VRG: 2.50 (2–2.5)	MMSE TG1: 27.00 (24.75–28.00) TG2: 27.00 (25.00–29.00) VRG: 27.00 (25.00–28.00) Education received (years): TG1: 8.00 TG2: 9.50 VRG: 8.00	Primary: 6 MWT Secondary: 10 MWT SRT Body mass index MPQ-39 WHODAS 2.0 GDS	T0 (baseline) T1 (after 8 weeks)	Compare the effects of functional training, cycling and exergaming on walking ability in elderly patients with PD	Exergame training has achieved similar results to traditional treatments in improving gait; all three strategies are recommended, considering patients' motivation
Gandolfi et al., 2017 [28] Virtual reality telerehabilitation for postural instability in Parkinson's disease: a multicenter, single-blind, randomized, controlled trial	RCT, 6	No: 76 VRG: 38 TG: 38 Age VRG: 67.45 TG: 69.84 Disease duration, (years) VRG: 6.16 TG: 7.47	VR nonimmersive	VRG: treatment with “Wii fit gaming system and balance board” at home, supervised via Skype by a physiotherapist TG: in-clinic sensory integration balance training (SIBT) 50′ treatment, 3 times a week for 7 consecutive weeks	I.C: age > 18; H&Y 2.5–3; stable therapy the month before; can perform postural transfer and stand for 10 minutes; presence of caregivers E.C: cardiovascular, orthopedic and otovestibular disorders; visual or neurological conditions interfering with balance; severe dyskinesia or on-off fluctuations; MMSE <24; depression	—	VRG: 2.5 TG: 2.5	MMSE VRG = 26.77 (1.48) TG = 28.64 (6.96)	Walking/balance Primary: BBS Secondary: ABC 10 MWT DGI MPQ-8	T0 (baseline) T1 (after treatment) T2 (after 1 month)	Compare the improvements in postural stability after balance training with VR at home remotely supervised and in-clinic. balance training with sensory integration	Use of VR at home (TeleWii) is a valid alternative to conventional rehabilitation to reduce postural instability in patients with PD (H&Y 2.5–3)
van den Heuvel et al., 2014 [29] Effects of augmented visual feedback during balance training in Parkinson's disease: a pilot randomized clinical trial	Pilot RCT, 8	No: 33 VRG: 17 TG: 16 Age: VRG: 66.3 (6.39) TG: 68.8 (9.68) Disease duration, (years) VRG: 9 TG: 8.8	Nonimmersive VR	VRG: interactive balance games with augmented visual feedback via an LCD monitor connected to a PC, a force plate, and an inertial sensor TG: conventional treatment 10 treatment sessions of 60′, for 5 weeks	I.C: H&Y 2–3; able to participate in both training programs; verbal and written informed consent E.C: other neurological, orthopaedic, or cardiopulmonary diseases that prevent participation in the study; MMSE <24; recent change in dopaminergic therapy; cognitive, visual or speech problems that prevent participation	—	VRG: 2.5 TG: 2.5	MMSE VRG: 29 TG: 28	Primary: FRT Secondary: BBS H&Y UPDRS (I, II, III, IV) FES MPQ-39 HAD Multidimensional Fatigue inventory	T0 (baseline) T1 (after 6 weeks) T2 (after 12 weeks follow up)	Investigate whether a balance training program that uses augmented visual feedback is feasible, safe, and more effective than conventional balance training in improving postural control in PD patients	The use of augmented visual feedback in a group setting is safe and feasible to provide therapeutic balance training for patients with PD, even if no more effective than conventional therapy
van der Kolk et al., 2019 [30] Effectiveness of home-based and remotely supervised aerobic exercise in Parkinson's disease: a double-blind, randomised controlled trial	RCT, 7	No: 130 VRG:65 TG:65 Età VRG: 59.3 TG: 59.4 Disease duration, (years) VRG: 3.41 TG: 3.16	Nonimmersive VR	VRG: aerobic pedalling exercises at home (30′–45′at least 3 times a week) enriched with virtual reality software and real-life videos to create an exergaming experience TG: stretching and relaxation (30′, 3 times a week)	I.C: H&Y>/= 2; practice less than the recommended physical activity for older adults; age 30–75; stable dopaminergic therapy E.C: beta-blocker or antipsychotic drugs; neurological, orthopaedic or heart problems; psychiatric illness; MMSE <24	Arthralgia/back pain (VRG = 2) Palpitations (VRG = 4)	VRG:4 TG: 3	MoCA VRG.: 26.3. (2.2) TG: 26.3 (2.5) Education received (years): VRG.: 15.1 (4.0) TG: 16.1 (4.5)	Primary: MSD-UPDRS III (off) Secondary: VO2 UPDRS III (on) UPDRS IV Number of falls, 6 MWT TUG mini-BEST Pegboard FTT MPQ-39 HADS SCOPA FSS TMT MoCA	T0 (baseline) T1(after 6 months)	Evaluate the effectiveness of aerobic exercise, gamified, and performed at home, to promote therapy adherence and relieve motor symptoms in patients with PD	The study provides level 1 evidence that aerobic exercise alleviates motor symptoms in Parkinson's disease and improves cardiovascular fitness
Liao et al., 2015 [31] Virtual reality-based training to improve obstacle crossing performance and dynamic balance in patieshewidmernts with Parkinson's disease.	RCT, 7	No: 36 VRG: 12 TG: 12 GC:12 Age VRG: 67.3 TG: 65.1 CG: 64.6 Disease duration, (years): VRG: 7.9 TG: 6.9 CG: 6.4	Nonimmersive VR	VRG: VR exercises with Wii Fit Plus games and Wii Fit balance board TG: traditional treatment (stretching, strengthening, balance) CG: fall prevention education 12 treatment sessions of 45 '(+15' treadmill in VRG and TG), 2 times a week for 6 weeks	I.C: H&Y 1–3; autonomous walking without aids; stable therapy; MMSE>/= 24 E.C: unstable medical conditions; other neurological, cardiopulmonary, orthopaedic diseases; history of seizures; pacemaker; visual impairment	No adverse events	CG:1.9 TG: 2 VRG: 2	MMSE VRG: 29.5 (0.7) TG: 29.8 (0.3) CG:29.7 (0.6)	Primary: performance overcoming obstacles with “Liberty system” Dynamic balance with “balance master system” Secondary SOT MPQ-39 FES-I) TUG	T0 (baseline) T1(after 1 day) T2(30 days after treatment -follow up)	Examine the effects of virtual reality-based exercise on overcoming obstacles in patients with Parkinson's	VR with Wii, as part of a multi-faceted workout, is effective in improving performance when overcoming obstacles, dynamic balance, functional capacity, and quality of life in patients with PD
Liao et al., 2015 (b) [32] VR-based Wii fit training in improving muscle strength, sensory integration ability and walking abilities in patients with MP	RCT, 7	No = 36 VRG: 12 TG: 12 GC: 12 Age VRG: 67.3 TG: 65.1 CG: 64.6 Disease duration, (years) VRG: 7.9 TG: 6.9 CG: 6.4	Nonimmersive VR	VRG: exercises with Wii Fit and treadmill TG: conventional training and treadmill CG: fall prevention education 12 sessions, twice per week, for 6 weeks	I.C.: H&Y 1–3; autonomous walking without aids; stable therapy; MMSE ≥ 24 E.C: unstable medical conditions; other neurological, cardiopulmonary, orthopaedic diseases; pacemaker;	—	CG: 1.9 TG: 2 VRG: 2	MMSE VRG: 29.5 TG: 29.8 CG: 29.7	Gait: GAITRite FGA Muscle strength: dynamometer Sensory integration skills: SOT	T0 (baseline) T1 (after 6 weeks) T2 (after 1 month-follow up)	Examine the effects of virtual reality-based training in improving muscle strength, sensory integration capacity and walking in patients with PD	Wii training is as useful as traditional training in improving outcomes, and these improvements have persisted for at least a month. It is therefore suggested that Wii training be implemented in patients with PD
Ma et al., 2011 [33] Effects of virtual reality training on functional reaching movements in people with Parkinson's disease: a randomized controlled pilot trial	Pilot RCT, 5	No: 33 VRG: 17 TG: Age VRG: 64.77 TG: 68.13 Disease duration, (years) VRG: 5.32 TG: 5.16	Immersive VR	VRG: reach 60 moving balls with your right-hand using VR system and polarized glasses TG: roll 60 wooden cylinders with your left hand.	I.C: H&Y 2–3; Age 50–75; stable therapy; MMSE ≥ 24. Normal sight and hearing; right-handed to self-assessment E.C: other neurological conditions besides PD; musculoskeletal disorders impairing UL movements;	Fatigue (VRG = 1)	VRG: 2 TG: 2	MMSE VRG: 27.24 (3.09) TG: 26.31 (2.52)	Success rates of the required task (catching the ball) Kinematic data	T0 (baseline) T1 (After treatment)	To investigate whether practising with virtual moving targets would improve motor performance in people with Parkinson's disease	A short training program with VR improved speed of movement and accuracy in reaching real fixed objects. However, the transfer effect was minimal in reaching real moving objects
Maidan et al., 2017 [34] Disparate effects of training on brain activation in Parkinson disease	RCT, 4	No: 34 VRG: 17 TG: 17 Age VRG: 71.2 TG: 71.5 Disease duration, (years) VRG: 7.9 TG: 11.6	Nonimmersive VR	VRG: VR associated treadmill TG: treadmill only 18 Sessions 3 times a week for 6 weeks	I.C: age 60–90; H&Y 1–3; ability to walk independently for at least 5 minutes; anti Parkinson therapy E.C: MRI contraindications; psychiatric comorbidities; MMSE <24; other neurological disorders besides PD; orthopaedic problems, unstable therapy	—	—	MMSE VRG: 27.8 (0.4) TG: 28.3 (0.5) MoCA VRG: 22.9 (0.9) TG: 21.9 (0.8) Global cognitive score VRG: 89.7 (2.7) TG: 88.4 (2.6) Attention VRG: 88 (4.2) TG: 84.1 (4.2) Executive functions VRG: 87.5 (2.2) TG: 83.4 (3.2)	fMRI assessment Step parameters MoCa Computerized cognitive test battery	T0 (baseline) T1 (after 7 weeks)	Compare the effects of treadmill training with virtual reality and treadmill training alone on brain activation in patients with Parkinson's disease	The results suggest that the task-specific exercise provided by VR led to experience-dependent neuroplasticity and reduced the usefulness of activating compensatory cognitive functions resulting in greater automaticity. Training with VR has improved both motor and cognitive aspects of the altered front-striatal circuit
Maidan et al., 2018 [16] Evidence for differential effects of 2 forms of exercise on prefrontal plasticity during walking in Parkinson's disease	RCT, 4	No: 64 VRG: 30 TG: 34 Age VRG: 70.1 TG: 73.1 Disease duration, (years) VRG: 8.9 TG: 9.7	Nonimmersive VR	VRG: treadmill training with virtual obstacles on a screen ahead TG: treadmill 45' treatment, 3 times a week for 6 weeks	I.C: age 60–90; H&Y 2–3; autonomous walking for at least 5 minutes; anti-Parkinson therapy E.C: psychiatric comorbidities; MMSE <24; performance-impairing neurological diseases; orthopaedic problems that could compromise walking; unstable medical conditions including cardiovascular instability	—	—	MMSE VRG: 28.2 (0.3) TG: 28.3 (0.3)	Deambulation (electronic gangway with pressure sensors) Prefrontal activation (functional near infrared spectroscopy—fNIRS)	—	Investigate whether the VR-paired treadmill and the treadmill alone differently affect prefrontal activation and whether this could explain the differences in fall rates after surgery	Providing a combined cognitive-motor training intervention may result in specific changes in prefrontal activation patterns that improve functional abilities, reduce falls and the risk of falling, which in turn could slow deterioration in patients with PD
Mirelman et al., 2016 [35] Addition of a non-immersive virtual reality component to treadmill training to reduce fall risk in older adults (V-TIME): a randomised controlled trial	RCT, 8	No: 302 (MP: 130) VRG: 154 TG: 148 Age (elderly and PD) VRG: 74.2 TG: 73.3	Nonimmersive VR	VRG: VR associated treadmill (elderly and with PD) TG: treadmill training 45' sessions, 3 times a week for 6 weeks	I.C: age 60–90; walking without assistance for at least 5 minutes; stable therapy in the previous month; 2 or more falls in the previous 6 months; clinical dementia rating scale = 0.5; H&Y 2-3; anti Parkinson therapy E.C: psychiatric comorbidities; history of stroke, brain damage and other neurological disorders; acute lower back pain; rheumatic or orthopaedic diseases; MMSE <24	Present but not related to the study	—	MMSE VRG: 27.8 (1.8) TG: 28.2 (1.7) Education received (years) VRG: 13.1 (4.0) TG: 12.9 (3.9)	Primary: rate of accidental falls Secondary: gait speed/variability 2 MWT SPPB NeuroTrax Corp SF-36	T0 (baseline) T1 (after training) T3 (after 6 months)	Test the hypothesis that a treadmill intervention combined with non-immersive virtual reality, to address cognitive aspects, safe walking, and mobility, would lead to fewer falls than treadmill training alone	In a heterogeneous group of elderly people at high risk of falls, treadmill training associated with virtual reality led to lower fall rates than training with treadmill alone
Pelosin et al., 2020 [36] A multimodal training modulates short afferent inhibition and improves complex walking in a cohort of faller older adults with an increased prevalence of Parkinson's disease	RCT, 4	No: 39 (PD: 24) VRG: 17 TG: 22 Age (elderly and PD) VRG: 73.3 TG: 71.9	Nonimmersive VR	VRG: treadmill training with obstacles and distractors in VR (elderly patients and with PD) TG: treadmill training 45′ treatment, 3 times a week for 6 weeks	I.C: 2 or more falls in the previous six months; age 60–85; walk for 5 minutes without assistance; H&Y 2-3; stable therapy for at least a month E.C: MMSE <24; psychiatric comorbidities; stroke or other neurological disease; contraindications to TMS; use of anticholinergics or acetylcholinesterase inhibitors	—	—	MoCA (elderly and PD) VRG 23.5 (4.3) TG: 25 (3.2) Education received (years) (Elderly and PD) VRG: 11.1 (4.2) TG: 9.8 (4.7)	Primary: Number of falls SAI magnitude Secondary: Gait parameters during normal walking (GaitRite) Overcoming obstacles	T0 (baseline) T1 (after 1 week) T2 (after 6 months)	Evaluate whether virtual reality-based attention training modulates cholinergic activity (SAI-short-latency afferent inhibition) and affects obstacle negotiation performance in a cohort of elderly people with a history of falls and with a higher prevalence of PD	The multitasking training carried out modulated the SAI and allowed functional improvements in gait. Furthermore, the combination of such rehabilitation approach with cholinergic pharmacological agents may optimize the recovery induced by the rehabilitation
Pompeu et al., 2012 [37] Effect of Nintendo Wii™-based motor and cognitive training on activities of daily living in patients with Parkinson's disease: a randomised clinical trial	RCT, 5	No: 32 VRG: 16 TG: 16	Nonimmersive VR	VRG: 10 games with Wii-Fit for motor and cognitive training TG: balance exercises without feedback or cognitive stimuli. 14 training sessions of 60′ (30′ stretching, strengthening + 30′ balance), 2 times a week for 7 weeks	I.C: age 60–85; H&Y 1–2; good visual and auditory acuity; 5–15 years of education; no other neurological or orthopaedic diseases; dementia (cut-off 23 MMSE) or depression (GDS cut-off 6) E.C: no other experiences in using the Wii fit; not having participated in other rehabilitation programs.	—	—	MoCA VRG: 20.6 (4.5) TG: 21.7 (4.6)	Primary: UPDRS II (ADL) Secondary: BBS UST MoCA	T0 (baseline) T1 (after treatment) T2 (after 60 days—follow up)	To study the effect of Nintendo Wii™-based cognitive-motor training compared to balance training on activities of daily living in patients with Parkinson's disease	Patients with PD showed better performance in daily life activities after 14 balance training sessions, without any additional benefits associated with motor and cognitive training with VR
Shih et al., 2016 [38] Effects of balance-based exergaming intervention using the Kinect sensor on posture stability in individuals with Parkinson's disease: a single-blinded randomized controlled trial	RCT, 6	No: 20 VRG: 10 TG: 10 Age VRG: 67.5 TG: 68.8 Disease duration, (years) VRG: 27.4 TG: 28.2	Exergaming/nonimmersive VR	VRG: balance training with exergaming (Kinect sensor) TG: balance training 50′ sessions (30′ balance), 2 times a week for 8 weeks	I.C: H&Y 1–3; MMSE ≥ 24; stable therapy; can stand without help E.C: history of other neurological, cardiovascular, orthopaedic disease related to postural instability; uncontrolled chronic diseases	—	VRG: 1.6 TG: 1.4	MMSE VRG: 27.4 (2.59) TG: 28.2 (1.99).	Postural stability: LOS OLS balance: BBS TUG	T0 (baseline) T1 (after 8 weeks)	Examine the effects of balance-based exergaming training using the Kinect sensor on postural stability and balance in people with Parkinson's	Balance training with exergame resulted in a greater improvement in postural stability than conventional training. The results support the therapeutic use of exergaming with Kinect sensor in patients with PD
Yang et al., 2016 [39] Home-based virtual reality balance training and conventional balance training in Parkinson's disease: a randomized controlled trial	RCT, 7	No: 23 VRG: 11 TG: 12 Age VRG: 72.5 TG: 75.4 Disease duration, (years) VRG: 9.4 TG: 8.3	Nonimmersive VR	VRG: At home. balance training with VR via Wii and balance board TG: conventional balance training at home. 12 sessions of 50′, twice a week, for 6 weeks	I.C: age 55–85; MMSE >24; H&Y 2–3; no balance or step training in the previous 6 months; no other clinical conditions related to balance or walking C: E: untreated depression; major visual/hearing impairments	No adverse events. 1 VRG patient dropped out as he preferred conventional training	VRG:3 TG: 3	MMSE VRG: 27.5 ± 4.0 TG: 27.2 ± 2.5	Primary: BBS Secondary: DGI TUG MPQ-39 UPDRS-III	T0 (baseline) T1 (after 6 weeks) T2 (after 8 weeks-follow up)	Assess whether virtual reality home balance training is more effective than conventional home balance training in improving balance, walking and quality of life in patients with Parkinson's disease	The results do not show significant differences in the improvements in balance and walking in the two treatment groups. In any case, exercises with VR at home can represent a valid alternative for patients with PD with limited access to rehabilitation services
Yen et al., 2011 [40] Effects of virtual reality-augmented balance training on sensory organization and attentional demand for postural control in people with parkinson disease: a randomized controlled trial	RCT, 7	No: 42 VRG: 14 TG: 14 GC: 14 Age VRG: 70.4 TG: 70.1 GC: 71.6 Disease duration, (years) |VRG: 6.0 TG: 6.1 GC: 7.8	Nonimmersive VR	VRG: balance training with dynamic balance board, LCD screen with 3D games (Virtools 3.5) TG: standing balance training. GC: no treatment. 30′ sessions, twice a week for 6 weeks	I.C: MMSE >24; H&Y 2–3; not having participated in other balance and gait training; ability to follow simple commands and the absence of chronic uncontrolled diseases E.C: history of other neurological, cardiovascular, orthopaedic diseases; on-off motor fluctuations and dyskinesia >3 on UPDRS	No adverse events, apart from the tendency to fall	VRG: 2.6 TG: 2.4 GC: 2.6	MMSE VRG: 28.5 (1.6) TG: 28.5 (1.2) GC: 28.1 (0.8)	SOT balance score	T0 (baseline) T1 (within 7 days after 6 weeks) T2 (after 10 weeks-follow up)	Examine the effects of balance training, associated with VR, on sensory integration of postural control and compare the results with those obtained from a conventional balance training group and an untrained control group	Both balance training with virtual reality and without could be considered valid for improving the sensory integration capacity for postural stability in people with PD

FOG: freezing of gait; VRG: virtual reality group; TG: treatment group; H&Y: Hoehn and Yahr scale; VR: virtual reality; MMSE: mini mental state examination; MoCA: montreal cognitive assessment; Mini-BEST: mini-balance evaluation systems test; NFOG-Q: new freezing of gait questionnaire; TMT-B: trail making test; SPPB: short physical performance battery; FSST: four square step test; FES-I: falls efficacy scale-international; PASE: physical activity scale for the elderly; UPDRS: unified Parkinson's disease rating scale; UL: upper limbs; MCI: mild cognitive impairment; BBS: Berg balance scale; TUG: timed up and go; FGA: functional gait assessment; 6 MWT: six minute walk test; SRT: sitting rising test; MPQ-39: multidimensional personality questionnaire; WHODAS 2.0: WHO disability assessment schedule; GDS: geriatric depression scale; ABC: activities-specific balance confidence scale; 10MWT: 10 meter walk test; DGI: dynamic gait index; FRT: functional reach test; HADS: hospital anxiety and depression scale; VO2max: maximum oxygen consumption; FTT: finger tapping test; SCOPA: scales for outcomes in Parkinson's disease; FSS: fatigue severity scale; SOT: sensory organization test; FMRI: functional magnetic resonance imaging; FNIRS: functional near infrared spectroscopy; 2 MWT: 2 minute walk test; SF-36: short form health survey 36; SAI: short-latency afferent inhibition; UST: unipedal stance test; LOS: limits of stability; OLS: one leg stand test.

**Table 2:** PEDro classification: methodological quality.

Author	1	2	3	4	5	6	7	8	9	10	11	Total score
Bekkers et al. [25]	N	Y	N	Y	N	N	Y	Y	N	Y	Y	6/10
Del Din et al. [26]	N	Y	N	Y	N	N	N	N	Y	Y	Y	5/10
Feng et al. [27]	Y	Y	N	Y	N	N	Y	Y	Y	Y	Y	7/10
Ferraz et al. [17]	Y	Y	Y	Y	N	N	Y	Y	N	Y	Y	7/10
Gandolfi et al. [28]	Y	Y	N	Y	N	N	Y	Y	N	Y	Y	6/10
van den Heuvel et al. [29]	Y	Y	Y	Y	N	N	Y	Y	Y	Y	Y	8/10
van der Kolk et al. [30]	Y	Y	Y	Y	N	N	Y	N	Y	Y	Y	7/10
Liao et al. [31]	Y	Y	Y	Y	N	N	Y	Y	N	Y	Y	7/10
Liao et al. [32]	Y	Y	Y	Y	N	N	Y	Y	N	Y	Y	7/10
Ma et al. [33]	N	Y	Y	Y	N	N	N	N	N	Y	Y	5/10
Maidan et al. [34]	N	Y	N	Y	N	N	Y	N	N	Y	N	4/10
Maidan et al. [16]	Y	Y	N	Y	N	N	Y	N	N	Y	N	4/10
Mirelman et al. [35]	Y	Y	Y	Y	N	N	Y	Y	Y	Y	Y	8/10
Pelosin et al. [36]	N	Y	N	Y	N	N	Y	N	N	Y	N	4/10
Pompeu et al. [37]	N	Y	N	Y	N	N	Y	N	N	Y	Y	5/10
Shih et al. [38]	Y	Y	Y	Y	N	N	N	Y	N	Y	Y	6/10
Yang et al. [39]	Y	Y	N	Y	N	N	Y	Y	Y	Y	Y	7/10
Yen et al. [40]	N	Y	N	Y	N	N	Y	Y	Y	Y	Y	7/10

Y = yes; 1. Eligibility criteria; 2. random distribution of subjects in each group; 3. secret allocation of subjects; 4. similar groups regarding the most important prognosis; 5. blind participation of subjects; 6. Blind participation of therapists; 7. blind examiners; 8. at least one key result obtained in more than 85% of subjects; 9. subjects received treatment or control condition; 10. intergroup statistical comparisons have been performed for at least one key outcome; 11. presence of precision and variability measures.

**Table 3:** Risk of bias of the included studies.

		Random sequence generation	Allocation concealment	Selective reporting	Blinding of participants and personnel	Blinding of outcome assessment	Incomplete outcome data	Other bias
Bekkers et al. 2020	High	?	−	+	−	+	−	?
Del Din et al. 2020	High	?	−	+	−	−	+	?
Feng et al. 2019	Low	?	−	+	−	+	+	?
Ferraz et al. 2018	Low	+	+	+	−	+	−	?
Gandolfi et al. 2017	Low	+	−	+	−	+	−	?
Heuvel et al. 2014	Low	?	+	+	−	+	+	?
Kolk et al. 2019	Low	+	+	+	−	+	−	?
Liao et al. 2015	Low	?	+	+	−	+	−	?
Liao et al. 2015 (b)	Low	?	+	+	−	+	−	?
Ma et al. 2011	High	+	+	+	−	−	−	?
Maidan et al. 2017	High	?	−	−	−	+	−	?
Maidan et al. 2018	High	?	−	−	−	+	−	?
Mirelman et al.2016	Low	+	+	−	−	+	+	?
Pelosin et al. 2020	Low	+	−	+	−	+	+	?
Pompeu et al. 2012	High	+	−	+	−	+	−	?
Shih et al. 2016	High	+	+	+	−	−	−	?
Yang et al. 2016	Low	+	−	+	?	+	+	?
Yen et al. 2011	Low	+	−	+	−	+	+	?

“+” means low risk of bias; “−” means high risk of bias; “?” means unclear risk of bias. Trials involving three or more high risks of bias were considered of poor methodological quality.

## Data Availability

The data supporting this systematic review are from previously published studies, which have been cited.
